# Site-Specific PEGylation of Therapeutic Proteins

**DOI:** 10.3390/ijms161025831

**Published:** 2015-10-28

**Authors:** Jonathan K. Dozier, Mark D. Distefano

**Affiliations:** 1 Department of Chemistry, University of Minnesota, Minneapolis, MN 55455, USA; E-Mail: dozie015@umn.edu; 2 Department of Medicinal Chemistry, University of Minnesota, Minneapolis, MN 55455, USA

**Keywords:** chemical modification, enzymatic modification, PEGylation, protein PEGylation, site specific modification, therapeutic proteins

## Abstract

The use of proteins as therapeutics has a long history and is becoming ever more common in modern medicine. While the number of protein-based drugs is growing every year, significant problems still remain with their use. Among these problems are rapid degradation and excretion from patients, thus requiring frequent dosing, which in turn increases the chances for an immunological response as well as increasing the cost of therapy. One of the main strategies to alleviate these problems is to link a polyethylene glycol (PEG) group to the protein of interest. This process, called PEGylation, has grown dramatically in recent years resulting in several approved drugs. Installing a single PEG chain at a defined site in a protein is challenging. Recently, there is has been considerable research into various methods for the site-specific PEGylation of proteins. This review seeks to summarize that work and provide background and context for how site-specific PEGylation is performed. After introducing the topic of site-specific PEGylation, recent developments using chemical methods are described. That is followed by a more extensive discussion of bioorthogonal reactions and enzymatic labeling.

## 1. Introduction

The use of proteins and peptides as therapeutics has a long and successful history. Starting with recombinant insulin in 1982, protein-based therapies have become an important tool in combating disease and illness with over 130 proteins and peptides approved for clinical use by the Food and Drug Administration (FDA) [1,2]. Due to their large size and specific conformation, proteins have the advantage of being highly specialized for their binding and/or activity. This means that there is less of a chance for cross reactivity, which can cause potentially fatal side effects. In addition, since most protein therapeutics are based on endogenously expressed proteins, there is less of a chance of developing an immunogenic reaction to them. In addition, because of their vast diversity, proteins can be used to treat a number of different illnesses: from treating endocrine disorders, to combating various cancers, to alleviating autoimmune diseases, to being the active agent in many vaccines. This diversity makes proteins attractive options for researchers to use in developing novel therapeutics [3].

However, using protein-based therapeutics is not without challenges—one of the drawbacks being their potentially short half-lives within the body. This is mainly due to degradation by endogenous proteases or by clearance of the protein from the body by the kidneys. This means that for many protein therapeutics, frequent dosing is necessary, which can cause complications such as eliciting an immune response [4,5,6] or negative side effects due to spikes in the protein level that lead to toxicity. Additionally, frequent dosing generally causes a drug to be at a competitive disadvantage compared to other therapies and also increases the likelihood of patient non-compliance during the dosing schedule. Another potential drawback for some protein therapeutics is there can be dose limiting solubility problems, which can prevent a protein’s use as a treatment.

**Figure 1:**
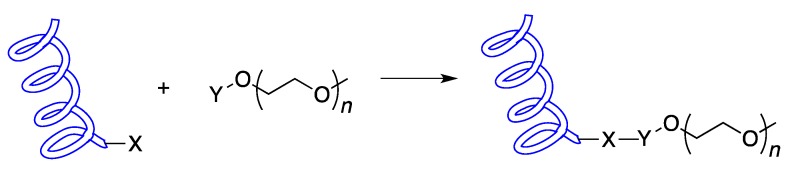
The general strategy for protein PEGylation: A functional group (X) on a protein is reacted with a complementary group (Y) on a poly(ethylene glycol) polymer (PEG) molecule forming a protein–PEG conjugate.

There have been a number of different strategies used for increasing a protein’s half-life in blood [7,8], including glycosylation [9], protein fusion [10] and albumin conjugation [11]. One of the most widely used solutions to overcoming these obstacles is to attach a poly(ethylene glycol) polymer (PEG) to the protein, a process known as PEGylation (See Figure 1).

The PEG moiety offers numerous advantages for increasing a protein’s stability and circulating half-life. Due to its flexibility, hydrophilicity, variable size, and low toxicity, PEGylation has been intensely studied in recent years as the method of choice for extending protein half-life. PEG has been approved by the Food and Drug Administration (FDA) as “generally recognized as safe” [12]. There are currently over 10 different PEGylated products currently approved by the FDA, with many more potential products in development (See Table 1). Even though the total number of PEGylated drugs is small compared to the total number of protein-based therapeutics on the market, a number of these PEGylated drugs are considered “blockbuster drugs” [13]. Overall, this suggests that there is a large potential for growth in commercial PEGylated therapeutics.

Early work with PEGylating proteins and enzymes came from the laboratory of Frank Davis and coworkers in the late 1970s [14,15,16,17]. Their method involved the nonselective modification of proteins by linking a methoxy–PEG group to the amino groups present on proteins using cyanuric chloride as the coupling reagent. They showed that PEGylated proteins had longer half-lives in the blood stream and decreased immunogenicity. These early studies demonstrated the value and utility of protein PEGylation.

Since these early studies in protein PEGylation, much research has been performed to maximize the advantages of PEGylation without incurring some of the disadvantages that result from modifying the protein’s structure. This work led to the first commercial PEGylated protein therapeutic, a PEGylated form of adenosine deaminase (ADA) [18,19]. ADA–PEG is used to treat severe combined immunodeficiency (SCID) in patients with a deficiency in the ADA protein due to an inherited genetic condition. The success of this therapy was followed by a number of different PEGylated protein therapeutics being approved by the FDA, including PEGylated Interferon-α2b (INF-α2b) and Interferon-α2a (INF-α2a), which are used for the treatment of Hepatitis B and C [20,21,22], l-asparaginase as part of a treatment for certain types of leukemia [23], and uricase that breaks down uric acid as a therapy for gout [24] (See Table 1).

Most of these therapeutic proteins rely on the non-specific PEGylation of the protein through reactions with the amino groups on the side-chain of lysines and the N-terminus. While this method is convenient for the creation of the PEGylated conjugates, it often leads to a heterogeneous mixture of PEGylated material with each PEG conjugate having its own activity and stability properties. For instance, the PEGylation of INF-α2a creates eight different PEGylated proteins through eight different lysine residues. The activity level of these different PEGylated isomers have a three-fold range between the most active isomer and the least active [25]. Similar results have been observed for PEGylated forms of INF-α2b [26], and a growth factor analogue [27].

Beyond potentially streamlining production and purification, an important feature of site-specific protein PEGylation is that it can result in minimizing the decrease in activity associated with PEGylation while retaining the pharmacokinetic benefits that accompany polymer attachment. While almost all PEGylated therapeutics show a decrease in specific activity compared to the non-PEGylated form [28], much more of the wild type activity could be retained if the site of PEGylation is controlled so that it creates a homogeneous product with the polymer attached to a residue in the protein that minimizes its effect on biological activity. This should have substantial therapeutic benefits because the protein retains much of its normal function, requiring lowing dosing and thereby minimizing side effects, while at the same time manifesting much higher circulating half-lives and thus requiring less frequent dosing which itself carries a number of significant advantages as was discussed previously. Overall, site-specific PEGylation of proteins has the potential to greatly enhance the use of proteins as therapeutics.

**Table 1:** Polyethylene glycol (PEG)-modified protein therapeutics approved by the food and drug administration (FDA). Site-specific PEGylated proteins given in bold [13,29].

Drug	Protein	Protein Size (kDa)	PEG Size (kDa)	Functional Group on PEG	Site of Attachment	Site-Specific	Year of Approval	Use	Reference
Adagen^®^	Adenosine deaminase	40	5	Succinimidyl ester	Lysines, serines, tyrosines, histidines	No	1990	Severe combined immunodeficiency disease	[18]
Oncaspar^®^	Asparaginase	31	5	Succinimidyl ester	Lysines, serines, tyrosines, histidines	No	1994	Leukemia	[23]
PegIntron^®^	Interferon-α-2b	19.2	12	Succinimidyl ester	Lysines, serines, tyrosines, histidines,cysteines	No, but 47.8% one isomer	2000	Hepatitis C	[26]
Pegasys^®^	Interferon-α-2a	19.2	40	Succinimidyl ester	Lysines	No	2001	Hepatitis C	[25]
Neulasta^®^	Granulocyte colony-stimulating factor (G-CSF)	18.8	20	Aldehyde	N-Terminal amine	Yes	2002	Neutropenia	[30]
Somavert^®^	Human growth hormone (hGH)	22	5	Succinimidyl ester	Lysines, N-terminus, phenylalanine	No	2003	Acromegaly	[31]
Mircera^®^	Erythropoietin	30 (18 unglycosylated)	40	Succinimidyl ester	Lysines	No	2007	Anemia	[32]
Cimzia^®^	Anti-tumor necrosis factor (TNF) α Fab’	51	40	Maleimide	C-Terminal cysteine	Yes	2008	Rheumatoid arthritis, Crohn disease, psoriatic arthritis	[33]
Krystexxa^®^	Urate oxidase	34	10	*p*-Nitrophenyl carbonate ester	Lysines	No	2010	Gout	[34]
Omontys	Synthetic, dimeric peptide (erythropoiesis stimulating agent)	4.9	40 (2 20 kDa chains)	Succinimidyl ester (added during chemical synthesis of the peptide)	Lysines	Yes	2012 (Recalled 2014)	Anemia in chronic kidney disease	[35]

While most PEGylated protein therapeutics are prepared via non-specific PEGylation, there are currently two commercially available therapeutics where the PEG chain has been introduced in a site-specific manner: a N-terminally PEGylated human recombinant granulocyte colony-stimulating factor (rh-GCSF), pegfilgrastin, marketed under the brand name Neulasta [30] and a thiol-PEGylated antibody fragment of the anti-tumor necrosis factor (TNF)-α monoclonal antibody, certolizamab pegol, sold as Cimzia [36].

Certolizumab pegol, marketed as Cizmia, is a highly successful site-specifically PEGylated protein therapeutic. Certolizumab is derived from an antibody fragment that recognizes tumor necrosis factor α (TNFα), which is partly responsible for the inflammatory effect for several autoimmune disorders [36]. While there are several other antibody based drugs on the market that target TNFα, including infliximab and adalimumab [37], certolizumab pegol is an Fc-free antibody fragment that targets TNFα and requires PEGylation in order to have a dosing schedule of every two weeks, which is similar to the full-size antibody drug targets of TNFα [38]. Additionally, certolizumab is the only commercially available PEGylated antibody or antibody fragment. It is PEGylated with a 40 kDa PEG moiety by reacting a C-terminal cysteine with a PEG–maleimide, creating a site-specific protein–PEG therapeutic [29].

Pegfilgrastin was developed from a previously available human recombinant protein therapeutic for rh-GCSF, called filgrastim, which was used in the treatment of neutropenia and its associated infections caused by chemotherapy or bone marrow transplantation [30]. rh-GCSF was shown to be site-specifically PEGylated at the N-terminus under mildly acidic conditions.[39]. This method was then used to create a PEG-rh-GCSF conjugate using a 20 kDa PEG–aldehyde molecule, where the aldehyde group was used for reductive amination of the N-terminal amine to form a secondary amine linkage between the PEG and the protein [29]; this conjugate became commercially available as Neulasta [40]. Studies showed that a single injection post-chemotherapy cycle with pegfilgrastin had comparable effects to daily injections post-chemotherapy with the non-PEGylated form of the protein [41,42]. Since then, Neulasta has become a “blockbuster” drug and in 2012, Neulasta was in the top 20 grossing prescription drugs globally, with $4.3 billion in total global sales [43].

Given the successes of drugs including Cimzia and Neulasta in conjunction with some of the problems with non-specific PEGylation, there is significant interest in the development of methodology for site-specific protein PEGylation. While there have been several excellent reviews of protein PEGylation in general [13,44,45,46,47], this article seeks to focus on recent efforts in the area of enzymatic site-specific PEGylation. In particular, the use of enzymes for site-specific modification which to date include Sortase, Protein Farnesyltransferase, Sialyltransferase, Tranglutaminase, and Formylglycine-generating enzyme. To set the stage for that, some of the chemical methods for creating site-specific PEGylated proteins are described to both highlight recent developments in the field and as way to better understand some of the enzymatic methods for creating PEG conjugates. That is important since enzymatic strategies are often combined with chemical methods to produce highly specific PEGylated therapeutic proteins. Overall, this review illustrates the therapeutic benefits of site-selective PEGylation, summarizes some key non-enzymatic methods, and finally describes numerous options for enzymatic site-selective modification.

## 2. Chemical Synthesis

One of the simplest ways to site-specifically modify a protein is through direct chemical synthesis where either the protein is generated via solid phase peptide synthesis (SPPS) and the PEG is incorporated in one of the coupling steps or through direct chemical attachment of a native chemical feature of the protein. SPPS incorporation has been used successfully in a number of cases. Jølck *et al.* incorporated an alkyne into a lipopeptide generated by SPPS and then used an azide–PEG molecule to attach the PEG group to the alkyne-containing residue using the Huisgen 1,3-dipolar cycloaddition, also known as the “click” reaction [48]. Subsequent removal of the other protecting groups and cleavage from the resin occurred under normal conditions making this method a convenient way to site-specifically install a PEG group on a peptide or protein that can be synthesized by SPPS.

Pandey *et al.* used site-specific SPPS to incorporate a PEG group on the WW domain of human Pin 1 [49]. This method incorporated the PEG group onto the growing peptide using an Fmoc-Asn(PEG)-OH residue that has been previously described [50]. They wanted to PEGylate this protein domain because its folding energy landscape is well characterized and it forms a β-sheet that is a common structural feature in many different proteins. Studying the effect of PEGylation on this small domain could help inform what happens when much larger proteins are PEGylated. Interestingly, the authors found that the conformation stability is dependent on the length of the PEG chain. While short PEG groups do increase the rate of folding, longer PEG groups not only increase the folding rate but also decrease the rate of unfolding. Their findings indicate that not only do PEG groups help stabilize protein folding, they also help prevent protein unfolding, which may help stabilize the protein–PEG complex *in vivo*.

Besides using SPPS to chemically label peptides or proteins, other chemical methods can be used as well. Hydrazine-containing PEG molecules can be used to form linkages with the oligosaccharides of glycoproteins [51]. In addition, as discussed earlier in reference to pegfilgrastin, proteins can be site-specific modified at their N-terminus by reacting the protein at slightly acidic pH with an aldehyde [39,52,53]. This strategy works by taking advantage of the fact that pK_a_ for the N-terminal α-amino group is approximately 7.8 whereas for the ε-amino group on lysine, the pK_a_ is 10.1. When the reaction is performed under acidic conditions, the lysine amine group is predominantly protonated and therefore unable to react with the aldehyde group, making the free amine on the N-terminus the only site for modification.

While many of these chemical methods maybe convenient for small polypeptides or proteins where the N-terminus is not involved in binding or activity, or in oligosaccharide containing molecules, more sophisticated methods are required to PEGylate the vast number of therapeutically relevant proteins.

## 3. Amino Acid Labeling

### 3.1. Cysteine Tagging

**Figure 2:**
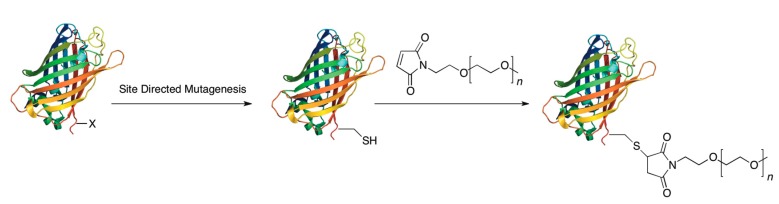
Site-specific PEGylation of a protein via chemical modification of cysteine: First a cysteine is encoded into a protein that lacks any free cysteines. Then, that cysteine is reacted with a maleimide–PEG group forming a covalent bond between the protein and the PEG group.

One of the most common methods for site-specific protein PEGylation is to genetically encode a single cysteine residue into a protein. This method works as a site-specific method for PEGylation because cysteine accounts for less than 1% of the total amino acid content of proteins and many of the cysteines found in proteins are involved in disulfide bonds making them unreactive to many thiol-specific reagents. The most common modification is reacting this free cysteine with a maleimide group attached to a PEG moiety (Figure 2).

Nidetzky and coworkers used this technique to create a PEGylated version of a l-lacate oxidase (LOX) from *Aerococcus viridans*, which is an enzyme that is used as a biological transducer for different biosensors [54]. They chose to mutate a serine residue that was believed to be sufficiently accessible enough for reaction with a maleimide PEG and because it was not a highly convserved residue as revealed in a comparison of different members of the α-hydroxy acid oxidases. Their results showed that the mutated protein had about half the activity of the wild type enzyme but upon PEGylation there was only a 30% decrease in activity. While both the PEGylated and unmodified mutant showed about 2.5-fold abated resistance to enzyme activation as compared to the wild type, it demonstrates the difficulty that even small changes in protein structure can cause. However, the fact that the enzyme still retains activity upon incorporation of a PEG makes it a candidate for biosensor immobilization.

Another recent use of adding an unpaired cysteine to create a site-specific PEG conjugate comes from the Yao lab [55]. They recently reported PEGylation work on glucagon-like peptide-1 (GLP-1), a promising therapeutic in combating diabetes. They conjugated the PEG group to the protein through a C-terminal cysteine using a 20K maleimide PEG derivative. They were able to show that the protein retained its original structure and that PEGylated protein had similar *in vivo* effects as the unmodified protein. Additionally, the PEGylated GLP-1 showed decreased blood glucose levels and higher insulin levels even after several hours post administration, which was not seen with the unmodified peptide. PEGylated GLP-1 also helped to reverse some of pancreatic tissue damage seen in diabetic mice. Through the C-terminal cysteine PEGylation, the authors were able to create a protein therapeutic that has applications to alleviate a number of conditions associated with diabetes. Such experiments show the simplicity and adaptability of site-specific protein PEGylation.

Additionally, the Pan group used the cysteine labeling method to create mutants of human thyroid stimulating hormone (rhTSH) [56]. They found that by mutating a glycine on the α subunit to a cysteine did not result in any significant loss of protein activity. This mutant also yielded the highest amount of PEGylated product after reaction with a maleimide PEG: 85% for the monoPEGylated product. While the mutation itself did not affect the protein’s binding to its receptor, they did find that there was a size-dependent decrease in protein receptor binding for the PEGylated product. Finally, they tested the effect of rhTSH on the levels of the thyroid hormone T4 *in vivo*. Rats were injected with either unmodified rhTSH or a PEG conjugate of their αG22C rhTSH mutant, in which case they used a 40 kDa PEG. Rats injected with the unmodified protein showed a rapid increase in the amount of T4 in their blood stream. However, the amount of circulating T4 tapered off very quickly for rats injected with the unmodified protein and there was significantly more T4 in the blood serum of rats injected with the PEG conjugate. This trend held true for rats injected with just the conjugated protein—even up to 72 h post injection. This is another exciting example of how site-specific PEGylation of proteins can be used to improve protein lifetime in the blood stream and help to improve polypeptides for use as therapeutics.

Site-specific cysteine labeling also offers the benefit of being able to easily change the site of modification to reduce unwanted site effects. The Lee lab has looked at how different sites of PEGylation affect a protein’s activity and the relationship between PEG conformation and activity [57]. In a 2012 paper, they examined the PEGylation of a small protein, Exendin-4 (Ex-4-Sys), using a combination of site-specific techniques to PEGylate the protein. The variant of the peptide they were using contained a C-terminal cysteine for which they were able to site-specifically PEGylate using a maleimide–PEG. Additionally, Ex-4-Cys was PEGylated performing a selective PEGylation of the N-terminus using a PEG–aldehyde under acidic conditions. Finally, Ex-4-Cys was non-specifically PEGylated using a succinimide PEG to conjugate the PEG group to lysine residues. This is non-specific because the protein contains two lysines, so they created a mixture of PEGylated products: PEGylation at K_12_, at K_27_ and at both K_12_ and K_27_. A cellular binding assay determined that the C-terminal cysteine PEGylation had the greatest receptor binding, most likely due to C-terminus being far removed from the receptor-binding site. Additionally, the use of a trimeric PEG group instead of a linear one also improved receptor binding as the trimeric PEG structure helps push the PEG group further away from the binding site of the receptor. The pharmacokinetic properties between the different PEG moieties did not vary greatly, but were significantly improved relative to the non-PEGylated form of the enzyme.

Another recent example of this technique was performed by Walker and coworkers to reduce vacuole formation that can be caused by PEGylated protein treatment [58]. Vacuole formation can potentially be a problem for people who are at a higher risk for renal failure. Recently, the relationship between the site of PEGylation and vacuole formation has been examined. Xu *et al.* used site-specific PEGylation to study how PEGylating FGF21, a protein that lowers glucose and increases insulin sensitivity, affects its activity and vacuole-forming properties. They created a library of 15 mono-PEGylated versions of FGF21 by creating site-specific mutations of cysteine at different amino acid residues within the protein and then conjugating them using a maleimide PEG group. They found that, in general, compared to N-terminal labeled FGF21, when the PEG group was linked to an internal residue, there was less of a decrease in the protein’s activity which was within four-fold of the unmodified FGF21. The results also showed that the closer the PEGylation site was to the carboxy terminus, the greater the decrease in activity was. These conjugates also manifested less vacuole formation in mouse models compared to a PEG-only control. The authors of this paper also examined how performing double PEGylation affected activity and vacuole formation. There was a decrease in activity for the doubly PEGylated variants but it was not so decreased as to render the protein ineffective as a therapeutic. Additionally, they found a dosing regimen in mice that both reduced glucose levels and decreased body weight without causing vacuole formation in mice. These low doses using the doubly PEGylated conjugates could be used to help prevent one of the negative side effects from PEGylation.

The introduction of cysteines to create site-specific PEG conjugates is also applicable to small peptides as well. The Klok lab developed small peptides based on the HIV-1 envelope glycoprotein gp41 which has been shown to inhibit virus-host cell membrane fusion [59]. They created a series of PEGylated peptides by adding a cysteine at either the N- or C-terminus or by changing various residues within the peptide sequence to cysteines. These thiols were then PEGylated by incubating the peptide with a PEG–acrylate to obtain the final product. Interestingly, when the peptide was PEGylated at the N- or C-terminus, there was a large decrease in inhibitory activity, but when the PEG group was added to an internal residue, only a small decrease in activity was observed. These PEGylated forms of the peptide also show about double the half-life compared to the wild-type peptide in a trypsin degradation assay. These types of experiments not only demonstrate that PEGylation can be used in small peptides but that even in these small molecules, the site of PEGylation is critical to maximize protein activity.

The site where the cysteine mutation is introduced is often very important in determining how much of the original activity of the protein is retained after PEGylation. Pan *et al.* used the cysteine mutation method to PEGylate tumor necrosis factor-related apoptsis-inducing ligand (TRAIL) by mutating an asparagine to a cysteine and then PEGylating it with a PEG–maleimide [60]. The asparagine they chose to mutate was a site that is potentially *N*-glycosylated. Because this site may be naturally post-translationally modified, using it for PEGylation may not interfere with the protein’s function. They found that the site-specific PEGylation at that site dramatically improved pharmacokinetic properties compared to the natural form of the TRAIL protein. Additionally, they compared the activity and pharmacokinetic properties of the cysteine-modified protein to a form of TRAIL that had been site-specifically PEGylated through the amine at the N-terminus using an aldehyde containing PEG performed under acidic conditions. The protein modified at the natural site of post-translation modification (asparagine) had an improved half-life; moreover, activity was greatly improved in an *in vitro* cell culture model and also in an *in vivo* xenograft mouse model.

All these examples highlight the versatility and ubiquity of using cysteine labeling to create site-specific PEG conjugates. While there are many advantages to using cysteine to site-specifically PEGylated proteins, number of disadvantages to this method also exists. Foremost is if the protein of interest contains more than one free cysteine residue, then this method is no longer site-specific and a heterogenous mixture of products can form.

### 3.2. Cysteine Disulfide (Cystine) Labeling

**Figure 3:**
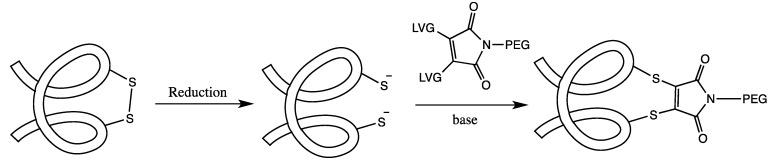
A general scheme for the PEGylation of disulfide bridges: The disulfide bond of the protein is first reduced and then both of the free cysteines are reacted with a bridging PEG-based reagent. This both attaches a PEG to the protein and helps to preserve the protein’s secondary structure.

A method related to labeling a single cysteine residue is to label a disulfide bond between two cysteines. The advantage to this approach *versus* using a cysteine mutation is that with the mutational strategy, there is the potential for protein dimerization or disulfide scrambling, the latter being especially significant in proteins with multiple cysteines. By labeling through cysteine bridges, many of these problems can be avoided because the disulfide bridges are usually native to the protein structure and many extracellular proteins, which are prime candidates for drug development, contain disulfide bridges. Using disulfides to attach a PEG group usually involves reducing the disulfide under mild conditions and then labeling the cysteines with a bis(thiol)-specific reagent (See Figure 3). While there is a risk of disulfide scrambling or protein aggregation with this method, these issues can be addressed by carefully monitoring the reaction, purifying out undesirable by-products, performing the reaction at low protein concentrations and selecting the best of the synthetic linker for the desired protein.

Balan *et al.* used a cysteine disulfide labeling method to site-specifically PEGylate both l-asparaginase and interferon α-2b (INF) [61]. They used a three carbon bridge consisting of an α,β-unsaturated β′-monosulfone dervitized with various PEG groups. Their experiments showed the l-asparaginase retained its biological activity but that INF had a large decrease in activity that was, unexpectedly, independent of PEG size. Other groups have sought to use bridging groups to covalently label disulfide bridges with PEG. Schumacher *et al.* used disubstituted maleimides with various leaving groups on the maleimide ring [62]. They found that they were able to effectively site-specifically label somatostatin with a dithiophenolmaleimide PEG group and that the PEGylated somatostatin retained its biological activity.

More recently, dithiomaleimides (DMTs) were used to create both PEGylated and fluorescent proteins and peptides [63]. O’Reilly and coworkers created a fluorescent and PEGylated form of salmon calcitonin (sCT), a small protein used in the treatment of several bone diseases. Initially they found that when they converted a dibromomaleimide (DBM) to DMT using mercaptoethanol, the product was highly fluorescent. This led them to label sCT using an *N*-poly(ethylene glycol)maleimide which reacts with the two cysteines in the protein normally bound in a disulfide bridge. In this way they were able to create a site-specific PEGylated protein that was also fluorescent. As these researchers have shown, using cysteine disulfide bridges to site-specifically label proteins can confer some unique advantages over the traditional single cysteine modification method and offers potential for the development of useful therapeutic agents in the future.

Other recent work has looked at developing new disulfide bridging systems that can work with antibodies and antibody fragments. Badescu *et al.* developed a new bis-alkylating reagent that can be used to label reduced disulfides in both antibodies and antibody fragments [64]. They demonstrated how they could label a cancer targeting antibody (trastuzumab) and its Fab fragment with a potent cytotoxic agent (monomethyl auristatin E) through the native disulfide bonds. Importantly, the conjugates were produced as a homogeneous product and retained their antigen targeting abilities. This is a promising new strategy for targeting proteins that contain intrinsic disulfide bonds and could potentially be used to PEGylate other proteins as well.

### 3.3. Tyrosine Tagging

While cysteine labeling is the most common canonical amino acid to be directly PEGylated, work has been done to explore other novel reactions with different amino acids. For example, Barbas and coworkers developed a novel reaction in which tyrosine reacts with a 4-phenyl-3H-1,2,4-triazoline-3,5(4H)-dione (PTAD) to create a covalent bond between a protein and molecule of interest [65]. Tyrosine is an attractive target because it is found at relatively low frequency in proteins and more importantly its use in conjugation does not require any prior reduction of the protein as is often necessary with cysteine modification. To employ this strategy for protein PEGylation, a PEG linked PTAD reagent was first prepared. They then reacted this with Chymotrypsinogen A, and compared those results with a sample of the same protein treated with an NHS–PEG reagent [66]. Because Chymotrypsin A has four tryrosine residues and fourteen lysine residues, it is theortetically possible to obtain a complex mixture of multiPEGylated products. For the NHS–PEG reaction, which should label on lysine, they observed a mixture of mono-, bis-, tri- and tetra-PEG species, but in the PTAD reaction, only a mono-adduct was observed. This result does not necessarily indicate that the reaction is site-specific, but rather highlights the increasing difficulty of coupling on additional PEG groups after the single addition of a PEG moiety to a tyrosine. However, these results are intriguing because they suggest that even in a protein containing multiple tyrosines, it may be possible to obtain a homogeneous product (due to differential reactivity) without any other modifications to the protein.

### 3.4. Serine and Threonine Reactions

While the hydroxyl group of serine or threonine is not sufficiently nucleophillic under most conditions to be useful for site-specific modification, selective reactions can be used to funcationalize these amino acids. Earlier work looked at targeting serines or threonines at the N-terminal position on proteins by using peroxiodate oxidation to generate a glyoxylyl group. The protein can then be reacted with an aminooxy containing PEG to create a covalent oxime bond. Gaertner *et al.* used this method to create a series of site-specific PEGylated proteins including IL-8, G-CSF and IL-1ra and showed that these proteins retained activity but had increased pharmacokinetic properties [67]. Other groups have looked at how the selective reaction of N-terminal serine residues (β-amino alcohols) with salicylaldehyde can be accomplished. When carried out using a PEG-functionalized salicylaldehyde, the protein can be selectively PEGylated on its N-terminus. Kirshenbaum and coworkers developed this methodology to create PEGylated versions of the S-protein (an important fragment of the RNase A) and a variant of parathyroid hormone (PTH 1-34) [68]. This approach offers a powerful alternative to cysteine-directed methods due to the simple and robust chemistry involved.

### 3.5. Histidine Tagging and Related Multiresidue Tags

**Figure 4:**
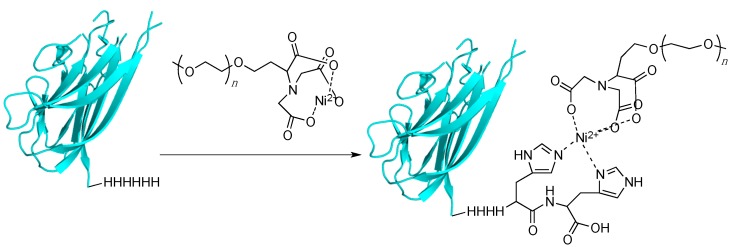
PEGylation using a His-tagging approach. A protein is encoded with a polyhistidine tag. Once incubated with a Ni–nitrilotriacetic acid (NTA)–PEG reagent, a complex is formed between the histidine residues and the nickel ion, thus PEGylating the protein.

For the past several decades, immobilized metal affinity chromatography has been used in the purification of a wide variety of proteins. The classic example of this is encoding a six-histidine tag onto the N- or C-terminus of a protein. This tag then complexes to a Ni(II) ion that is bound via nitrilotriacetic acid (NTA) resin. Proteins which lack the His-tag are washed away and the protein is eluded from the resin with high concentrations of imidazole to disrupt the histidine-Ni(II) binding interaction [69,70]. While this technique has been used extensively for protein purification, it has been less widely explored as a means of labeling proteins to enhance their therapeutic value; recently, several groups have begun to examine whether His-tags can be used to create site-specific PEGylated conjugates; in such a strategy, the His-tag would bind to Ni-NTA attached to a PEG moiety (Figure 4).

Kim *et al.* recently reported fusing tumor necrosis factor-related apoptosis inducing ligand (TRAIL) with a six-residue histidine tag and then labeling that protein with 5 kDa PEG containing a terminal nickel-NTA moiety [71]. They found that PEGylation gave a higher yield of the final product when the PEG group was functionalized with two Ni-NTA moieties rather than one. Once the PEGylation was complete, they discovered that the PEGylated material had a lower activity compared to the non-PEGylated version. However, the PEGylated form had improved pharmacokinetic properties such as half-time and AUC parameters. Finally, mice containing HCT116 human colon cancer tumor were injected with unmodified TRAIL and different TRAIL–PEG conjugates. The PEGylated TRAIL with two Ni-NTA groups significantly slowed the growth of the tumor compared to the unmodified protein. This method is convenient since a wide variety of proteins already carry a histidine tag for purposes of purification, so using this tag to also PEGylate the protein is convenient in that it does not require additional protein modification.

While this type of histidine tag has the advantage of being easy to use and site-specific, it lacks the irreversible character of a covalent bond and thus may lead to PEG release. This may be an advantage if the protein-substrate/receptor interaction is greatly perturbed upon PEGylation; however, having a weakly linked polymer could cause premature disassociation between the protein and PEG group and thus render the protein susceptible to normal excretion and degradation pathways.

Brocchini and coworkers used the His-tagging approach to PEGylate both a domain antibody (dAb) and interferon α-2a but using a covalent bond by conjugating the His-tag to a PEG-*bis*-sulfone [72]. Such sulfone-based reagents are capable of undergoing *bis*-alkyation with the nitrogens on the imidazole ring of histidine to create a covalent conjugate. This is similar to the chemistry used to create PEGylated conjugates via disulfide bond reduction and alkylation but instead of the two cysteines reacting, it is two histidine residues. They used this method to create PEG conjugates of a domain antibody for tumor necrosis factor α (dAb-TNFα) and interferon-α-2a (INF-α-2a), where the dAb-TNFα contained a 6-histidine tag on the C-terminus and the INF-α-2a contained an 8-histidine tag on the N-terminus. Since these proteins contain more than one set of two histidines, they needed to tailor the reaction to produce the mono-PEGylated species by controlling the reaction pH and concentration of the reactants. Alternatively, they were able to purify some of the diPEGylated species, which may be useful for creating different PEGylated products of the the same protein to test for differences in activity and pharmacokinetics. They found that the PEGylated dAb-TNFα retained about 90% of its activity in a TNFα-mediated cellular cytotoxicity assay. Additionally, the half-life in blood circulation of the PEGylated products was greatly increased compared to the non-PEGylated sample: the half-lives for non-PEGylated, mono-PEGylated sample and diPEGylated proteins were 5 min, 4.8 and 18 h, respectively. For IFN-α-2a, the PEGylated polypeptides showed an increase in their ED_50_ values from 7 pg/mL for the non-PEGylated protein to between 50–300 pg/mL for the monoPEGylated species, and 370–720 pg/mL for the diPEGylated species. The half-lives however for IFN-α-2a were substantially increased with greater PEG length. Non-PEGylated IFN-α-2a had a half-life of 1.2 h, an INF–20 kDa–PEG conjugate had a half-life of 13.3 h, a diPEGylated version of INF-α-2a with two 20 kDa PEG groups had a half-life of 25.4 h, and 40 and 60 kDa single conjugates had half-lives of 34.1 and 49.3 h, respectively. This work highlights the versatility that can be achieved by using His-tagging to create novel covalently modified site-specific PEG therapeutics. While histidine tagged proteins are not yet FDA approved from human therapeutics, demonstrating the utility and versatility of this tag for PEGylating therapeutics could lead to swifter approval and/or the development of other similar, yet regulatory acceptable, tags.

Beyond histidine tagging, other, longer, protein tags can be used for site-specific PEGylation. An example of this is the use of an intein fusion protein, that when derivatized with hydrazine produces a protein hydrazide. That functionality can be selectively reacted with PEG groups containing a ketone or aldehyde to produce a stable hydrazone bond. Thom *et al.* used this method to create C-terminal PEGylated versions of the therapeutically relevant IFN-α2a and INF-β2b [73].

## 4. Bio-Orthogonal Labeling

One novel way to site-specifically attach a PEG group onto a protein is to first incorporate a bio-orthogonal functional group into the protein; that is to incorporate functionality that does not occur naturally in cellular systems, and once a bio-orthogonal functional group is installed in the protein, a PEG molecule containing a complementary functional group for the moiety installed on the protein can then be reacted together to form a new covalent bond between the PEG group and the protein. Such functionalities include azides, alkynes, aldehydes, aminooxys, functionalized arenes, and trans-cyclooctenes. The challenge in biological chemistry is finding an appropriate method to site-specifically incorporate these chemical moieties [74]. While there are a number of different approaches to incorporating them into proteins, only a fraction have been used to create therapeutic protein–PEG conjugates. This type of PEGylation strategy remains an attractive option, though, because it results in a single product due to the bio-orthogonal functional group being present at only one specific location within a protein. This results not only in a homogeneous product, but also prevents off-target reactions in case any impurities remain in the sample. This type of labeling strategy has great potential for creating PEGylated biologics although currently, there are no approved products on the market created via this strategy.

### 4.1. Incorporation via Auxotrophic Strains

One way to incorporate bio-orthogonal functionality into proteins is to express the protein with a non-natural amino acid (NNAA) that contains such a bio-orthogonal functional group. One way to incorporate NNAAs into proteins is to use an auxotrophic strain of bacteria to express the protein. Auxotrophic bacteria are bacteria that cannot naturally produce a certain amino acid and must be grown in media supplemented with the amino acid. This works for the incorporation of NNAAs because once the bacteria have grown to a critical concentration in natural amino acid supplemented media, the media can then be exchanged for a media supplemented with the NNAA instead of the natural amino acid and the protein expressed. If the NNAA is similar enough in structure, para-azido phenylalanine for phenylalanine as an example, then the NNAA will be incorporated anywhere in the protein that would normally contain the natural amino acid. If the protein only has one of those amino acids in its primary sequence, then it becomes a site-specific incorporation. Methionine is the most frequently used because it is one of the least common amino acids [75].

This technique was used by Cazalis *et al.* to introduce an azido–methionine onto the C-terminus of a thrombomodulin derivative [76]. Once the protein was synthesized with the site-specific non-natural amino acid, it was reacted with a methyl-5k-PEG-triarylphosphine via the Staudinger ligation. They tested their PEGylated thrombomodulin derivative along with the non-PEGylated version and a commercially available thrombomodulin. The results showed that PEGylation had no effect on the protein’s activity compared to both the unmodified and commercially available versions.

A similar strategy was used to PEGylate another therapeutically relevant protein except at the N-terminus. Nairin *et al.* used an auxotrophic strain of *Escherichia coli* (*E. coli)* to incorporate an azide containing the residue, azidohomoalanine, on to the N-terminus of interferon β-1b (IFNb) [77]. Through site-specific PEGylation of IFNb, they minimized the likelihood that the protein’s activity would be compromised since the site of PEGylation can be controlled and chosen to minimize the PEG group’s interference with protein-receptor binding. Nairn *et al.* used a methionine auxotrophic strain of *E. coli* to incorporate the azide-containing amino acid because IFNb contains no methionines except for the N-terminal initiation site. Once the azide-containing IFNb was generated, they explored different reaction conditions under which the conjugation between the protein and alkyne containing PEG group could be performed through a “click” reaction. They found that by using DTT as the reducing agent they could perform the conjugation reaction at a 2:1 ratio of PEG to protein and that this condition gave the highest conversion to a final PEGylated product. Interestingly, the *in vitro* activity of the conjugate decreased with increasing PEG size as compared to a commercially available form of INFb, but that the PEG group increased both the total exposure and elimination half-life of the IFNb *in vivo.* To test PEGylated IFNb’s potential use as a therapeutic, SCID mice containing Daudi tumors were used to compare the PEGylated and non-PEGylated proteins. IFNb and its PEG conjugates were administered once a week for nine weeks. The tumor size was measured to determine whether IFNb could inhibit tumor growth. Importantly, using INFb equipped with a branched 40 kDa PEG group almost no tumor growth following weekly injections was observed compared to significant tumor growth for the non-PEGylated protein. When the injections were reduced to every other week for a PEGylated IFNb, the results showed that there was still a more significant decrease in the rate of tumor growth using PEGylated IFNb containing a branched 40 kDa PEG moiety, even when compared to the rate of tumor growth for mice injected three times a week with the commercially available (non-PEGylated) form of IFNb.

### 4.2. Nonsense Suppression Methods

Beyond using auxotrophic strains of bacteria, which is limited in terms of its site-specificity because the protein must either contain only one of the targeted amino acids or—if there are multiple residues—then only one must be accessible for conjugation, researchers have also used the amber suppression technique to install site-specific NNAAs for PEG conjugation. Amber suppression works by chemically acylating tRNA that is complementary to the UAG stop codon with an NNAA [78]. This tRNA-NNAA complex is then injected into cells that express the mRNA that encodes the target protein which has a UAG codon (installed via site directed mutagenesis) at the position where the NNAA is to be inserted. The cell’s own translational machinery then incorporates the NNAA into the growing polypeptide chain, yielding the desired protein. This technique has been used to create a number of protein conjugates, including protein PEG conjugates [75]. A recent article by Kim *et al.* summarizes the current state of genetically encoded unnatural amino acids and their use in protein conjugation [79]. Here we review only the more recent work using NNAAs to create protein–PEG conjugates.

Schultz and coworkers have worked extensively to develop this method, expanding it to work completely inside *E. coli* by engineering a tyrosyl tRNA/tRNA synthetase pair to selectively incorporate azido-phenylalanine into proteins. This bypasses the need to chemically acylate the tRNA with the NNAA. The *E. coli* can simply be incubated with azido-phenylalanine and the engineered tyrosyl tRNA/tRNA synthetase pair incorporates the NNAA into the protein [80]. This method was used to create an azide containing human superoxide dismutase-1 (SOD) and then conjugated it to an alkyne containing PEG reagent via the “click” reaction [81]. They showed that they could obtain site-specific incorporation of both a 5 and 20 kDa PEG group, and they observed no loss of function between either the azido modified enzyme or the PEG-conjugated enzyme compared to the wild-type enzyme.

More recently, Davis and coworkers have shown that proteins can be site-specifically PEGylated via Suzuki-Miyaura coupling using the nonnatural amino acid encoding method to incorporate either a *p*-iodo-benzylcysteine into a 3-layer-α/β-Rossman-fold protein or a *p-*iodo-phenylalanine into the all-β-helix protein 275–276 [82]. Afterward, they performed the Suzuki-Miyaura coupling using a palladium catalyst with pyrimidine and guanidine based ligands, and found that they could incorporate either 2 or 20 kDa PEG groups into both proteins using phenylboronic acid-based reagents. Interestingly, even in the absence of any ligand to promote catalysis, the reaction still occurred with a yield of greater than 90%, suggesting that the PEG–phenylboronic acid molecule may act as the ligand as well as the conjugation substrate. Other studies have shown that PEGylation can be accomplished using alkene pyrrolysine-containing NNAAs combined with thiol-containing PEGs. Such groups can undergo covalent bond formation upon UV irradiation. This was carried out to PEGylate an *E. coli* acid-chaperone protein (HdeA) and Asparaginase II [83].

Additional recent work using these developed NNAA-tRNA pairs have resulted in the construction of a system that can incorporate a *p-*acetylphenylalanine amino acid into a target protein of interest followed by conjugation to a 30 kDa PEG–oxime. Proteins as diverse as human growth hormone (hGH) and Fibroblast growth factor 21 (FGF21) have been PEGylated and shown to be therapeutically revelant using this technology [84,85].

These results highlight how NNAA can be a valuable tool in the development of protein–PEG conjugates, especially for installing chemical moieties, such as iodo-arenes, that are difficult to incorporate using other methods. The drawback to using this technique is that it either requires injecting the cell with the chemically acylated NNAA–tRNA complex or developing an orthogonal tRNA/tRNA synthetase pair to generate the NNAA–tRNA complex, both of which are highly time and labor intensive. Development of versatile NNAA–tRNA complex systems has been achieved by companies including Ambrx, which can greatly reduce the amount necessary to develop a new system but this technology must be licensed from the company potentially increasing the overall price of the therapeutic.

## 5. Enzymatic Labeling

Another method for introducing bioorthogonal chemical moieties into proteins, which can then be used to covalently PEGylate them, is enzymatic labeling. This technique uses an enzyme, which typically recognizes a specific amino acid sequence that can be introduced into a target protein, to catalyze the reaction between the protein of interest and a substrate analogue containing a bio-orthogonal functional group. This technique works best for enzymes that manifest promiscuous substrate specificity since it allows alternative substrates, which incorporate non-natural chemical groups, to be used without having to engineer the enzyme to accept them. A number of different enzymes have been used for this purpose including sortase, biotin ligase, lipoic acid ligase, formylglycine generating enzyme, sialyltransferases, phosphopantetheinyltransferase, galactose oxidase, prenyltransferases, transglutaminase and myristoltransferase [86]. While not all of them have been used for protein PEGylation, they all offer potential as PEGylation agents for therapeutic proteins. This section focuses on efforts where enzymatic labeling has been used to site-specifically PEGylate proteins.

### 5.1. Sortagging

**Figure 5:**
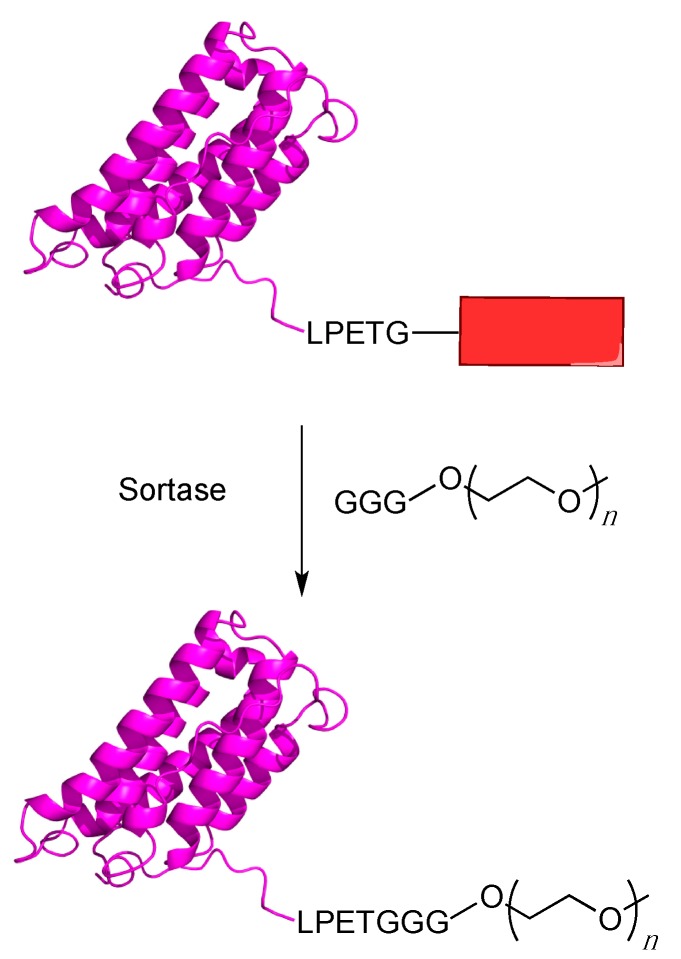
Site specific PEGylation using sortagging. A protein of interest is appended with a LPETG peptide sequence followed by any other desired sequence (red box). The sortase enzyme will then cleave the T–G bond and attach the triglycine PEG substrate to the protein via formation of a new amide bond.

The use of the sortase as a protein labeling reagent has grown increasingly popular in recent years. This enzyme catalyzes a transpeptidase reaction between an N-terminal amino group derived from glycine and a specific internal amino acid sequence on a protein, usually LPXTG. This results in the formation of a covalent bond between the C-terminal portion of the amino acid sequence and the formerly N-terminal glycine and is often called sortagging [87]. One of the most common sortase enzymes used is Sortase A (SrtA) derived from *Staphylococcus aureus* (*S. aureus)*, which recognizes the sequence LPETG and cleaves the peptide between the threonine and glycine residues creating a thioester bond with a cysteinyl thiol positioned in the active site of the SrtA enzyme. This is followed by an aminolysis reaction with the amino group of the N-terminus of a polyglycine moiety, thereby creating a new peptide bond between the protein and the polyglycine containing molecule. In the case of protein PEGylatation, the protein of interest is appended with LPETG tag (hence the name “sortagging”) and reacted with a polyglycine-derived molecule appended with a PEG group [88] (Figure 5).

There have been several examples of the use of SrtA for sortagging proteins. Early PEGylation work with sortase showed that eGFP bearing a C-terminal LPETG could be PEGylated with a 10 kDa PEG group linked to a triglycine moiety [89]. The results showed that eGFP was effectively PEGylated with the triglycine PEG and also, interestingly, PEGylation still occurred even when PEG contained only a simple amine group. This result suggests that a glycine residue is not strictly required for PEGylation using sortagging, making the PEG substrate much easier to obtain because it reduces the complexity of synthesis.

Ploegh and coworkers have been active in using sortagging for a number of applications, including PEGylation [90]. They have used sortase to PEGylate a form of IFN-α2a containing a C-terminal LPETG sequence. They PEGylated the protein by incubating the engineered INF-α2a with SrtA and a GGGK peptide containing either a 10 or 20 kDa PEG group affixed to the ε-amine group of lysine. They observed only a minor increase in the IC_50_ values for PEGylated INF-α2a as compared to both the unmodified protein and a wild-type protein standard. Additionally, they tested this method against G-CSF and found that LPETG tagged G-CSF could be modified with their 10 kDa PEG. This modification had only a small effect on the protein’s EC_50_, raising it from 2.5 pg/mL for the unmodified protein to 3.5 pg/mL for the PEGylated version. They found that for both proteins, the PEGylated forms had significantly higher circulatory half-lives than for their non-PEGylated counterparts. These results show that sortagging is a viable method for the creation of site-specific PEGylated protein therapeutics because while the tagged and PEGylated proteins do not have significantly decreased activities, they do have a much higher half-life in the blood stream.

While sortase has been used directly in PEGylation, Leung *et al.* recently reported creating protein delivery vehicles using PEG/poly(*N*-vinyl pyrrolidine) (PVPON) to create capsules that could be functionalized with a protein coating [91]. To functionalize the capsules, they incorporated an alkyne in the polymer chain and then used “click” based chemistry to attach an azide–PEG–GGG molecule to add a polyglycine moiety to the vehicle. Once the surface was coated with the triglycine moiety, they used sortase to immobilize a single-chain variable fragment (scFv) containing the LPETG tag to the polymer capsule. Those capsules were used to coat thrombin derived from blood platelets. This method is intriguing because the PEG group was used for stability purposes, but more importantly, for its ability to form biological compatible capsules.

This trend of using sortase mediated PEGylation for labeling large macroscopic particles with PEG-stabilized proteins has recently been used to label the surface of cells with proteins [92]. This method uses a specialized trifunctional labeling molecule with a lipid tail to bind into the cellular membrane, a 4 kDa PEG intermediate section to help solubilize and stabilize the protein, and a triglycine head for attachment to the protein. Tomita *et al.* used this method to first attach eGFP containing a LPETG sequence near its C-terminus to HeLa cells. By treating the cells with the GGG–PEG–lipid reagent and then incubating those cells with eGFP–LPETG and sortase, they were able to fluorescently label the surface of the HeLa cells. This same method was used to label E.G7 cells, a murine thymoma cell line, with the Fc domain of either IgG_1_ or IgG_2a_. Once they confirmed that this method would label both cell surfaces with the desired protein, they examined the phagocytosis rate in E.G7 cells co-cultured with immature dendritic cells. They observed that the cells labeled with the Fc domain for IgG_2a_ had a much faster phagocytosis rate than for cells labeled with the IgG_1_ Fc domain. These results confirmed what was previously known about the properties of the two antibody subclasses because IgG_1_ has a greater affinity for FcγRs, a receptor found on dendritic cells, which when bound to the IgG domain should decrease the rate of phagocytosis. Thus, using their site-specific PEGylation and anchoring method they could modulate cell–cell interaction. This type of research shows that site-specific PEGylation can also be used for fundamental biological research, and is not limited to making more effective therapeutics.

Another interesting application for protein PEGylation is through sortase-catalyzed reaction between an LPETG-containing protein and a hydrazine containing peptide. It was found that sortase will utilize hydrazine and hydrazine containing molecules in place of the triglycine substrate to create C-terminally labeled proteins. Li *et al.* used this methodology to create azide and alkyne C-terminally labeled proteins (Ubiquitin and eGFP), which they subsequently PEGylated with a corresponding PEG derivative [93]. Thus, while there are no approved PEGylated therapeutics derived from sortagging to date, the availability of substrates and relative simplicity of the labeling method makes this a very attractive method for future use.

### 5.2. Protein Farnesyltransferase

Another useful enzyme for site-specific PEGylation of proteins is Protein Farnesyltransferase (PFTase). PFTase catalyzes the attachment of a farnesyl moiety from farnesyl diphosphate (FPP) to a cysteine reside four amino acids from the C-termini of proteins when it is present in a “CaaX-box” sequence where C is cysteine, a is an aliphatic amino acid, and *X* is one of a variety of amino acids [73,93,94]. PFTase is a convenient labeling reagent because it will tolerate both a number of different “CaaX-box” sequences as well as modifications to the terminal isoprenoid unit of FPP. PFTase has been used to label proteins with a number of different FPP analogues including azides and alkynes [95,96], for the click reaction; aldehydes and ketones for aminooxy conjugation [97,98]; NBD [99] and anthranilate [100] for fluorescence studies; and benzophenones [101,102] and diazotrifluoropropanoyl groups [103] for photolabeling.

**Figure 6:**
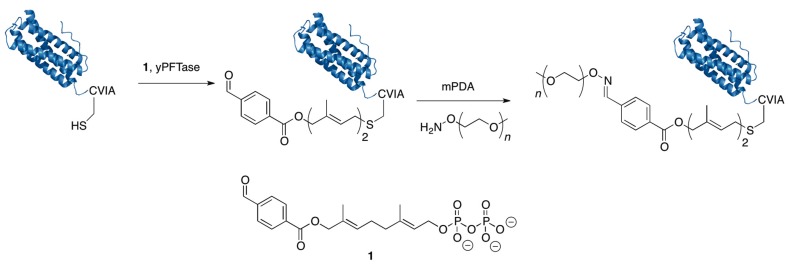
Protein PEGylation using protein farnesyltransferase (PFTase): A protein is engineered with a “CaaX-box” tag and then PFTase is used to attach a farnesyl aldehyde analogue to the cysteine of the CaaX box. Afterward, a secondary reaction is performed with an aminooxy–PEG group to form an oxime linkage between the protein and the PEG group. The catalyst *m*-phenylenediamine (mPDA) was used in these experiments.

Distefano and coworkers have used PFTase as a labeling reagent to create a number of different protein conjugates. To accomplish this, several novel FPP analogues for bioconjugation including analogues containing azides and alkynes [95,96] or aldehydes have been developed [97]. They showed how eGFP could be PEGylated by genetically incorporating a CVIA tag at the C-terminus, prenylating it using an aldehyde containing FPP analogue and then conjugating the modified protein to an aminooxy-10 kDa PEG molecule via an oxime ligation reaction [104] (Figure 6).

Additionally they showed that the eGFP can be rapidly purified and PEGylated by first prenylating eGFP with an aldehyde-containing substrate in the cell lysate and then immobilizing it using hydrazine-containing agarose beads. After washing the beads to remove unwanted proteins, the eGFP is released from the beads by reacting it with an aminooxy–PEG reagent which forms a more stable oxime bond compared to the hydrazone bond between protein and the beads. Thus, this results in the simultaneous purification and PEGylation of eGFP. Beyond this example, several other proteins have been PEGylated using the PFTase labeling method. Rashidian *et al.* showed it could be used to PEGylate Glucose-Dependent Insulinotropic Polypeptide (GIP), a small protein (~7 kDa) that has been investigated for the treatment of type 2 diabetes. One of the main disadvantages of using GIP as a therapeutic is that it has a short circulating half-life. GIP modified with a mini-PEG has shown resistance to proteolytic degradation while preserving its activity. Rashidian *et al.* showed that GIP encoded with a C-terminal CVIM tag could be PEGylated with a 5 kDa PEG molecule using their PFTase based strategy on a purified sample of GIP or using their rapid purification and PEGylation method on protein.

To improve this method based on aldehyde-containing substrates, Distefano and coworkers have investigated alternative catalysts to increase the rate of the oxime-forming PEGylation reactions. This research led them to identify *m*-phenylenediamine (mPDA) and *p*-phenylenediamine (pPDA) as more efficient catalysts for transoximization reactions [83,98]. They used mPDA to PEGylate eGFP by performing in-lysate prenylation followed by capture and release strategy from hydrazine beads as described above; the presence of the new catalyst substantially decreased the elution time in the PEGylation/release step. They used this same catalyst to PEGylate Dihydrofolate reductase (DHFR) conjugated to a ketone group incorporated by nonsense suppression with *p*-acetyl phenylalanine; DHFR is being investigated for a number of different applications in drug delivery [105,106]. Rashidian *et al.* were able to increase the PEGylation rate of DHFR using the mPDA catalyst by 2.5-fold compared to aniline. Recently, protein engineering of PFTase has been used to increase the efficiency of alternative substrate utilization including the aldehyde substrate used to introduce aldehyde functionality for subsequent PEGylation [107]. Overall, using PFTase as a site-specific enzyme for protein labeling offers several advantages in that it utilizes a small tag (four residues), can be used with a number of different bio-orthogonal reactions and is an efficient enzyme. These advantages should help make PFTase labeling an important tool for the creation of PEGylated therapeutics in the future.

### 5.3. GlycoPEGylation

Even the best-designed tagging strategies can sometimes inhibit a protein’s function. One of the ways to avoid this problem is to use an enzymatic post-translational modification that naturally occurs within a protein to install a PEG moiety. A common post-translational modification is glycosylation where a saccharide group is covalently transferred onto a protein. Several important therapeutic proteins, including granulocyte colony stimulating factor (G-CSF), interferon-α2b (IFN-α2b), and granulocyte/macrophage colony stimulating factor (GM-CSF), are naturally glycosylated via a hydroxyl group on either an internal serine or threonine, in a process referred to as *O*-glycosylation.

**Figure 7:**
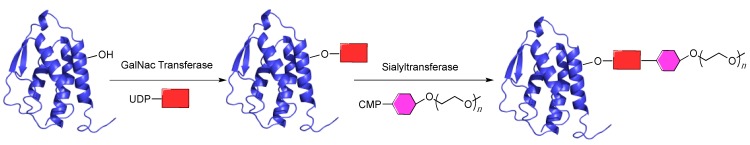
GlycoPEGylation of proteins: A protein that is naturally glycosylated is incubated with a galactosyltransferase and uridine 5′-diphospho-*N*-acetylgalactosamine (UDP-GalNac). GalNac is represented by a red box. Once the GalNac is installed on the protein, it is next incubated with a cytidine monophosphate (CMP) activated sialic acid group containing a PEG moiety (shown as pink hexagon) and a sialyltransferase. The enzyme adds the sialyl–PEG group to the GalNac residue and thus the protein becomes covalently PEGylated.

DeFrees *et al.* showed that they could PEGylate G-CSF, GM-CSF and INF-α2b using several different strategies [108]. This first involved identifying the glycosyltransferases that are responsible for modifying these different proteins. Short peptide sequences based on the internal protein glycosylation sites for G-CSF, GM-CSF and INF-α2b were screened against a panel of different glycotransferases and used to identify several different *N*-acetylgalactosaminyltransferases (GalAc-T) that could efficiently glycosylate the three proteins. They initially glycosylated the proteins to form *O*-linked glycans that were subsequently glycosylated using a sialyltransferase to covalently attach a sialic acid residue containing a PEG group (Figure 7).

This method allowed for site-selective modification because of the specificity of the initial glycosylation reaction. As noted above, since the PEG group is attached to a position that is normally modified with a glycan, it minimizes the probability that PEGylation will result in a loss of activity.

The PEGylated versions of G-CSF and INF-α2b were evaluated for both pharmacokinetic properties and bioactivity. With INF-α2b, the PEGylated form showed a significantly lower clearance rate than the non-PEGylated protein and the former retained wild-type-like biological activity. Interestingly, when they tested the G-CSF protein, they compared it not only to the unmodified version, but also to a G-CSF conjugate that had been PEGylated through chemical modification of the amino terminus. They found that both PEGylated versions of G-CSF had a significantly lower (five-fold) clearance rate than the non-PEGylated version of G-CSF and with the glycoPEG version having a slightly higher (1.8-fold) area under the curve dosing profile compared to the chemically PEGylated version. The glycoPEG G-CSF also showed the greatest activity at simulating white blood cell production after injection into mice, exceeding that obtained using the chemically PEGylated version. These results indicate that using natural glycosylation sites on proteins for enzymatic PEGylation manifests all the advantages of standard PEGylation methods but eliminates some of the negative effects that result from modifying a protein at a site that is typically not modified in nature.

GlycoPEGylation has also recently been used in the development of a treatment for hemophilia since several coagulation factors currently in use are naturally glycosylated. Initial work examined how PEGylation can be used on coagulation factors that contain *N*-linked glycans. Stennicke *et al.* prepared a glycoPEGylated version of coagulation factor VIIa (FVIIa) [109]. This method involves producing and purifying the protein from mammalian cells and then removing the natural sialic acid moiety with a sialidase followed by treatment with a sialyltransferase and a PEG-containing sialyl analogue. Using this strategy, FVIIa proteins modified with a range of different PEG groups from 2 to 40 kDa were produced. It was found that while PEGylation caused only a small reduction in the protein’s activity, it does extend the time that the protein interacts with its cognate cell surface receptor, which could allow for an extended half-life in the circulation. Later work found that PEGylation had very minimal change on the structure of the protein by comparing coagulation factor FVIIa to PEGylated FVIIa and also revealed that PEGylation modestly increased the protein’s thermal stability [110]. This work was expanded to create a glycoPEG form of coagulation factor IX (FIX) by Østergaard *et al.* [111]. PEGylated FIX showed minimal perturbation in activity compared to the unmodified protein, but remarkably improved half-life in animal models, increasing from 16 h for the unmodified protein to 113 h for the PEGylated protein. This glycoPEGylated protein is currently being investigated for therapeutic use in clinical trials [112].

This desialylation/resialylation strategy for PEGylation has also been studied for *O*-linked modifications [113]. Turoctocog-α, a recombinant form of coagulation factor VIII (FVIII), which is used as another treatment for hemophilia, has a unique *O*-glycan on its B-domain suitable for glycoPEGylation. As noted above, this PEGylation strategy requires production of the protein in mammalian cell culture followed by sialidase treatment to remove the natural sialyl groups. The protein is then PEGylated by incubating it with a PEG–sialic acid analogue and a sialyltransferase, (ST3GalI in this case), which specifically transfers the synthetic analogue to the *O*-glycan site. After PEGylation, the protein is incubated with a sialic acid substrate and a different sialyltransferase, ST3GalIII, which reattaches the sialic acid moieties back onto the *N*-glycan sites. This *O*-glycoPEGylated FVIII showed a two-fold prolonged half-life in animal models, and similar potency and efficacy as compared to the unmodified protein.

Recently, Park *et al.* used a combination of sialic acid and galactose-mediated chemistry to glycosylate thyrogen (rh-TSH) and then used either a chemical or enzymatic method to add an aldehyde group to the protein, which was then used to conjugate an aminooxy–PEG group [114]. This method, like the previous glycosylation methods, has the dual advantages that it is both site-specific and that the site of PEGylation occurs at a site of normal modification; hence it is less likely to interfere the protein’s activity. Those investigators showed that upon PEGylation using the glycolysation/PEGylation method, rh-TSH showed decreased activity as both the size of the PEG group and number of sites of PEGylation increased. However, the IC_50_ values of all PEG conjugates were within 10-fold of the unmodified protein, meaning they retained significant function. Additionally they found that using this PEGylation method, rh-TSH conjugated with 40 kDa PEG groups, had a 23-fold increase in plasma half-life compared to unmodified rh-TSH. They also showed that in a mouse model, the T_4_ thyroid hormone level rapidly rose with injection of the unmodified rh-TSH protein but that circulating T_4_ was no longer detectable after 48 h. In contrast, mice injected with rh-TSH PEG conjugates, containing either a single 40 kDa PEG group or several10 kDa PEG groups, showed increased levels of the T_4_ hormone even after 72 h post-dosing for both conjugates.

Overall, using glycotransferases to PEGylate proteins is an attractive strategy for the site-specific modification of proteins containing natural glycosylation sites. It offers the ability to create a homogeneous product like other site-specific PEGylation strategies but also has the advantage of being much less disruptive to natural function of the protein because the site of PEGylation is normally modified in the natural system. Based on the favorable results with coagulation factors, glycoPEGylated therapeutics show considerable promise.

### 5.4. Transglutaminase

**Figure 8:**
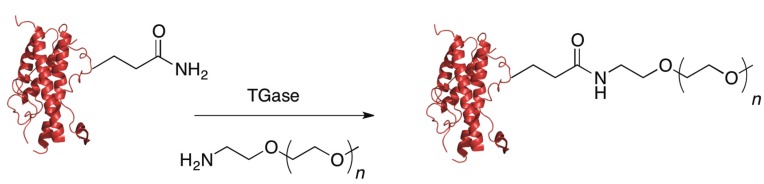
Transglutaminase (TGase) catalyzed PEGylation: A protein with a glutamine residue in a flexible loop is incubated with a TGase and a PEG molecule containing a primary amine. The TGase will PEGylate the protein, creating a new covalent bond.

Another class of enzymes that has frequently been used for protein labeling are the transglutaminases (TGases). These enzymes create a covalent bond between a primary amine, normally the ε-amino group from lysine, and the carboxamide group of glutamine via an acyl transfer reaction; the glutamine residue must be in a flexible loop portion of the protein in order for the reaction to occur. Transglutaminases are attractive options for protein PEGylation because they are rather promiscuous in the primary amine they will accept as substrates, so as long as the PEG group contains a primary amine, it will most likely be a substrate for the enzyme (Figure 8).

Due to this promiscuity, transglutaminases have been used to create a number of different therapeutically relevant protein conjugates derived from human growth hormone (hGH), salmon calcitonin [115], interleukin-2 [116,117], and apomyoglobin [118]. There are several very thorough reviews covering the use of transglutaminases to modify proteins [119,120,121]. The focus here is on the development of PEGylated therapeutics and strategies used to site-specifically PEGylate proteins with TGase.

Pasut and coworkers have studied potential differences between chemical PEGylation *versus* enzymatic PEGylation with TGase [122]. In particular, they examined how the properties of PEGylated human growth hormone (hGH), a therapeutic protein used for the treatment of a number of endocrine conditions, differed depending on whether it was generated by chemical modification at the N-terminus or enzymatically using transglutaminase. A PEG aldehyde was used to selectively label the N-terminus of the protein. As noted earlier, when performing the chemical reaction at pH 5, preferential labeling of the N-terminal amine group occurs because all the ε-amino groups on lysine side-chains will be protonated. Thus, selective N-terminal PEGylation of hGH was performed using this approach. In the same study, enzymatic labeling of hGH using TGase and a PEG reagent incorporating a primary amine was carried out. Interestingly, even though hGH carries 13 glutamine residues, 63.3% of the reaction product was a monoPEGylated form at position 141. This illustrates that while TGase may be promiscuous in terms of the amine substrate it will accept, the enzyme is partially selective in terms of the carboxamide substrate; the microenvironment of the glutamine residue is critical in determining its reactivity. After preparing the chemically and enzymatically PEGylated material, the authors studied the pharmacokinetics of the PEGylated hGH and compared them to the unmodified protein by administering the proteins intravenously to rats and analyzing hGH levels in blood plasma. They found that the pharmacokinetics were greatly improved for both types of PEGylated proteins and that the site of PEGylation did not significantly change the pharmacokinetic parameters. However, the *in vivo* potency of the modified proteins was not determined and may vary depending on the site of PEGylation.

One of the main problems involved with using TGase to PEGylate proteins is that it is difficult to predict which glutamine residue(s) will become labeled. One important aspect of TGase research has been the development of methods to identify which glutamines become PEGylated by TGases and determine whether a particular type of TGase is specific for a given protein. Sato *et al.* showed the effective use of a microbial TGase; they could site-specifically modify interleukin-2 with a sugar moiety as well as a PEG moiety [117]. Mero *et al.* developed an MS screening method to identify the specific residues that became PEGylated using microbial TGase with a low molecular weight monodisperse PEG [118]. They tested this method with several different therapeutically relevant proteins including G-CSF, hGH and apomyoglobin.

Beyond screening for the site of PEGylation, Maullu *et al.* developed a computational model to attempt to identify which glutamines would become PEGylated by bacterial TGase [123]. They developed a computer model which correctly predicted that only one glutamine (Q134) out of a possible 17 sites on G-CSF becomes PEGylated using microbial TGase. This led them to formulate some specific conclusions concerning the site of PEGylation: the site should be solvent exposed and in a flexible region, a proline close to a glutamine reduces the probability it will be PEGylated, and that with glutamines close to each other it is probable that only one will be PEGylated as steric hindrance of the newly attached PEG group will prevent PEGylation of the other site.

An alternative strategy to control TGase reactivity is to engineer new variants of the TGase enzyme that are site-specific. One way to accomplish this is to screen a library of different transglutaminases to identify variants that are site-specific [124]. Zhao *et al.* developed a high-throughput transglutaminase assay that measures the amount of a radiolabeled putrescine incorporated into a protein substrate. They used this method to screen mutants of microbial TGase for site-specificity against human growth hormone that contains two glutamines (Q40 and Q141) that normally undergo labeling with the wild-type enzyme. Using this approach, a TGase mutant that specifically labeled Q141 was identified. While they used this method to find one specific mutant for hGH, it is theoretically applicable for any protein. Overall, the largest obstacle for using TGase for enzymatic labeling concerns the inability to predict and control glutamine reactivity. However, the development of computational and screening methods have proved useful in addressing this problem.

### 5.5. Formylglycine-Generating Enzyme

Formylglycine-generating enzyme (FGE) catalyzes the oxidation of an internal cysteine residue to formylglycine, thus installing an aldehyde group within the protein [125]. Berotizzi and coworkers recently developed an FGE consensus sequence, CXPXR, that is recognized by the enzyme [126]

**Figure 9:**
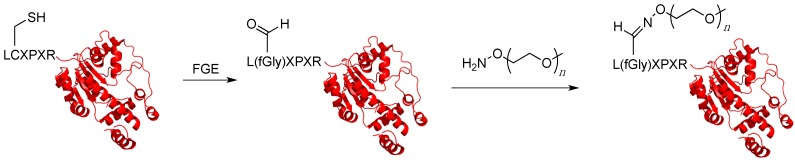
PEGylation using formylglycine-generating enzyme (FGE): FGE will convert a cysteine in a CXPXR consensus sequence to an aldehyde-containing formylglycine (fGly) residue. This new aldehyde can then be used to react with an aminooxy-containing PEG molecule to create an oxime bond between the PEG and the protein.

. They were able to add this sequence to the N-terminus of a mycobacterial sulfotransferase and the C-terminus of maltose binding protein; FGE-catalyzed reactions with these engineered proteins allowed conversion to formylglycine and subsequent PEGylation with aminooxy containing PEGs ranging in molecular weight from 5 to 20 kDa (Figure 9). The short sequence recognized by FGE makes its use for protein PEGylation an attractive option.

## 6. Perspectives and Conclusions

The site-specific PEGylation of proteins offers a number of unique advantages over non-specific strategies for protein modification including increases in activity over non-specific labeling, enhanced stability of the protein, and longer *in vivo* half-lives. The number of different techniques developed for this purpose has continued to evolve and new, and ever more sophisticated methods are being developed at an accelerated frequency. While many of the techniques mentioned in this review are generally considered site-selective there is always the possibility of side reactions or other unforeseen interactions, which can cause unwanted labeling of protein by a PEG group. These types of adverse effects are more likely to occur in methods that target native chemical moieties such as cysteine labeling or conjugation through the N-terminal amine at acidic pH and even in the case of promiscuous enzymatic reactions such as TGase. Solid phase PEGylation or PEGylation through a non-natural amino acid offer the most selective means of PEGylation and even enzymatic reactions that recognize specific amino acid tags, such as PFTase and Sortase, offer very high degrees of selectivity. One of the biggest challenges with protein PEGylation involves optimizing a PEGylation strategy that is specific to the target protein of interest.

The evolution of site-specific protein PEGylation strategies has been driven by an increasing demand for site-specifically PEGylated proteins. While there are several hundred approved protein therapeutics currently in use, there are only a few produced in PEGyated form and just two in which the PEG group has been introduced in a site-specific manner. The paucity of site-specifically PEGylated drugs, despite all their potential advantages, offers tremendous opportunities for growth in the field. Developing new site-specific PEG-conjugates is attractive because unmodified forms of many therapeutic proteins and PEG itself are already approved by regulatory agencies for use in humans; this potentially simplifies the approval process for new PEGylated products based on existing therapeutics and could result in accelerated timelines from research to market.

Currently, there are two main research directions in the field of protein PEGylation research. One involves applying existing strategies to previously non-PEGylated proteins while the other is focused on the development of new PEGylation methodologies. The most common approach is to employ established site-specific PEGylation methods, cysteine modification being most precedented, and apply this to a protein of interest. While cysteine labeling with a maleimide PEG has the advantages that it is well-established, easy to perform and PEG maleimides are commercially available, it does require the protein to have one and only one free cysteine in order to be site-specific. This limits its applicability and requires structural information to insure that if the protein does contain multiple cysteines, these residues are either involved in disulfide bonds or sufficiently shielded within the protein’s core so as to not react with the PEG maleimide.

The use of non-natural amino acids offers an intriguing possibility for the development of PEGylated protein therapeutics, especially given the commercialization of these technologies from companies including Ambrx, which offer NNAA/PEG pairs that can be used in a wide variety of proteins. However this method is still limited its expense and also because it still requires screening the protein for the best position in which to make the mutation.

Enzymatic site-specific modification is a major growth area in the PEGylation field. While there are numerous site-specific enzymatic labeling methods including sortagging, biotin ligase tagging, lipoic acid ligase labeling, formylglycine generating enzyme labeling, sialylation, phosphopantetheinyltransferase labeling, galactose oxidation, prenylation, transglutamination and myristolylation, only a handful of these methods have been employed to prepare site-specific PEG conjugates. Currently, there are no approved products that use enzymatic PEGylation with one major drawback being the difficulty in scaling up many of these reactions for commercial production. However, given their mild reaction conditions, availability of substrates and rapid kinetics, enzymatic labeling methods appear primed to enhance the field of protein PEGylation for the development of therapeutic proteins with new and improved properties.
